# A Novel HRAS Mutation Independently Contributes to Left Ventricular Hypertrophy in a Family with a Known MYH7 Mutation

**DOI:** 10.1371/journal.pone.0168501

**Published:** 2016-12-21

**Authors:** Maria Elena Sana, Lawrence A. Quilliam, Andrea Spitaleri, Laura Pezzoli, Daniela Marchetti, Chiara Lodrini, Elisabetta Candiago, Anna Rita Lincesso, Paolo Ferrazzi, Maria Iascone

**Affiliations:** 1 USSD Laboratorio di Genetica Medica, Azienda Socio Sanitaria Territoriale Papa Giovanni XXIII, Bergamo, Italy; 2 FROM Research Foundation, Azienda Socio Sanitaria Territoriale Papa Giovanni XXIII, Bergamo, Italy; 3 Department of Biochemistry and Molecular Biology, Indiana University, Indianapolis, Indiana, United States of America; 4 CONCEPT Lab, Istituto Italiano di Tecnologia, Genova, Italy; 5 USC di Anatomia Patologica, Azienda Socio Sanitaria Territoriale Papa Giovanni XXIII, Bergamo, Italy; 6 Centro per la Cardiomiopatia Ipertrofica e le Cardiopatie Valvolari, Policlinico di Monza, Monza, Italy; Universidad de Buenos Aires, ARGENTINA

## Abstract

Several genetic conditions can lead to left ventricular hypertrophy (LVH). Among them, hypertrophic cardiomyopathy (HCM), caused by mutations in sarcomere genes, is the most common inherited cardiac disease. Instead, RASopathies, a rare class of disorders characterized by neuro-cardio-facial-cutaneous abnormalities and sometimes presenting with LVH, are caused by mutations in the RAS-MAPK pathway. We report on a 62-years-old male who presented isolated severe obstructive LVH but did not carry the sarcomere mutation previously identified in his affected relatives. By exome sequencing, we detected a novel mutation in *HRAS* gene (NM_005343.2:p.Arg68Trp), present also in the proband’s daughter, who showed mild LVH and severe intellectual disability. The cardiac phenotype was indistinguishable between family members carrying either mutation. *In silico* studies suggested that the mutated HRAS protein is constitutionally activated. Consistently, functional characterization *in vitro* confirmed elevated HRAS-GTP accumulation and downstream RAS-MAPK pathway activation that are known to drive cell proliferation in LVH. Our study emphasizes the role of RAS signaling in cardiac hypertrophy and highlights the complexity in differential diagnosis of RASopathies. In fact, the mild features of RASopathy and the recurrence of sarcomeric HCM in this family delayed the correct diagnosis until comprehensive genetic testing was performed.

## Introduction

Left ventricular hypertrophy (LVH) is a condition characterized by myocardium thickening of the left ventricle in the heart that can be associated with different clinical conditions [1]. Hypertrophic cardiomyopathy (HCM) (MIM# 192600) is a common genetic cardiovascular disease with a prevalence of about 0.2% in the general population [2], characterized by primary unexplained LVH associated with nondilated ventricular chambers. The most common genetic cause of HCM are mutations in genes that encode elements of the sarcomere and cytoskeleton in cardiomyocytes, with *MYBPC3* (MIM* 600958) and *MYH7* (MIM* 160760) mutations accounting for one fourth to one third of all cases [3]. Metabolic, mitochondrial and infiltrative storage disorders can also clinically mimic HCM. Differential diagnosis is important since the mode of inheritance, natural history and treatment of these diseases are different from those of classical sarcomeric HCM [4].

Besides HCM and its clinical phenocopies, several rare syndromic conditions, particularly RASopathies, can present with LVH. These developmental disorders are caused by elevated Ras/mitogen-activated protein kinase (RAS-MAPK) pathway signaling [5] and include Costello syndrome (MIM# 218040), neurofibromatosis type I (MIM# 162200), Noonan syndrome (MIM# 163950), cardio-facio-cutaneous syndrome (MIM# 115150) and LEOPARD syndrome (MIM# 151100). Individuals with these rare syndromes are usually diagnosed during infancy and show variable degrees of intellectual disability, short stature, macrocephaly, hair and skin abnormalities, and facial dysmorphisms. Among other cardiac features, a subset of patients presents LVH [6].

Here we report the coexistence in an Italian family presenting with obstructive LVH of both sarcomeric HCM and a mild form of RASopathy caused by a novel *HRAS* (MIM* 190020) mutation. Comprehensive genetic and functional studies have allowed a clinical diagnosis.

## Materials and Methods

### Clinical data

An Italian family (5 members) with LVH was studied. The study was approved by the Institutional Ethics Committee "Comitato etico della provincia di Bergamo" and written informed consent was obtained from all participants. A comprehensive cardiovascular evaluation was performed on all patients, including a complete M-mode-2D and color-Doppler study to analyze cardiac chamber dimensions, global systolic and diastolic function, valve and great arteries anomalies. The diagnosis of LVH was established on the echocardiographic demonstration of interventricular septal and/or posterior wall thickness in end-diastole ≥15 mm in absence of other causes of LVH [7]. Global systolic function was assessed measuring fractional shortening in M-mode.

### Candidate mutation analysis

Since a *MYH7* mutation was previously identified in a relative of our proband, exon 23 and flanking regions were analyzed by Sanger sequencing in all remaining family members using BigDye Terminator 3.1 chemistry (Applied Biosystems, Foster City, California).

### Targeted exome sequencing

Next-generation sequencing was carried out on the proband. In solution hybridization capture of 159 cardiac genes was carried out using Trusight Custom Enrichment kit (Illumina, San Diego, California) according to manufacturers’ protocols. Probes for enrichment were designed using DesignStudio (Illumina, San Diego, California). The genes included in targeted resequencing panel were selected using “cardiomyopathy” and “arrhythmia” as keywords in OMIM database (OMIM®. World Wide Web (URL: http://omim.org) [August, 2013 accessed]) and HGMD^TM^ Professional database [8]. An enriched fragment library was sequenced by 2x100 bp sequencing protocol on a MiSeq sequencer (Illumina, San Diego, California).

### Data analysis

The Illumina Pipeline software with default parameters was used for image acquisition, image processing and signal processing. Data filtering and analysis were performed using a custom pipeline as previously reported [9, 10]. The set parameters for the variant calling were mapping quality of 20, minimum Phred base quality score of 30 and minimum read depth of 10X. Splicing variants were identified in a region ±10 bp from exon boundaries. The evolutionary conservation of affected nucleotides was evaluated by PhyloP score [11], and the effect of amino acid substitutions based on chemical properties, including polarity and molecular volume was assessed by Grantham score [12]. Disease-causing potential was evaluated by prediction tools: PolyPhen-2 [13], SIFT [14], MutationTaster [15]. Intronic and synonymous variations that were not located closest to splice sites were excluded from further analyses. The remaining coding and splice site variations were compared with publicly available variant databases, namely: 1) dbSNP Release 144 [16] 2) 1000 Genome Project (browser.1000genomes.org/); 3) NHLBI GO Exome Sequencing Project (ESP6500 data release) (Exome Variant Server, NHLBI Exome Sequencing Project (ESP), Seattle, WA (URL: http://snp.gs.washington.edu/EVS/) [December, 2015 accessed] and 4) Exome Aggregation Consortium (ExAC), Cambridge, MA (URL: http://exac.broadinstitute.org) [December, 2015 accessed]. Genetic variants found in any of these databases with a minor allele frequency (MAF) >0.01% were excluded from further analyses.

### Validation and segregation

Variants found by exome sequencing were validated and tested for segregation in the family by Sanger sequencing on an ABI PRISM 3130 DNA sequencer (Applied Biosystems, Foster City, California) using standard procedures.

### Molecular dynamics simulations

Molecular dynamics (MD) simulations were performed on HRAS wild-type (HRAS-wt) and HRAS mutant, each in complex with guanosine-5'-triphosphate (GTP) (PDB code: 1NVV [17]) and with guanosine 5′-diphosphate (GDP) (PDB code: 1Q21 [18]) using the GROMACS 4.6.2 package [19]. The GTP and GDP ligand partial charges and parameters were previously determined using the general Amber force field (GAFF) [20]. The topology were constructed as reported in [21] and then converted in GROMACS topology [22]. To probe the dynamic structural characteristics during the simulations, some quantities were computed. The Cα-RMSDs (root mean square deviations) were calculated from the minimal deviations of the Ca atoms of the trajectories away from the energy minimized structure with parallel organizations by superimposing the conformations. The molecular mechanics/generalized Born surface area (MM/GBSA) free energy was calculated as previously described [23]. The contact network was generated to analyze the intermolecular interactions by using an in-house python script.

### Generation and analysis of mutant HRAS

The transition NM_005343.2:c.202C>T was created in codon 68 of the *HRAS* cDNA by PCR and confirmed by Sanger sequencing. HRAS-Arg68Trp, HRAS-Gln61Leu and wild-type HRAS were subcloned into a modified pQCXI plasmid (Clontech Labs, Mountain View, California) encoding a hemagluttinin (HA) tag. To measure RAS activity, the above plasmids were transfected into HEK 293T cells using X-treme GENE HP reagent (Roche Diagnostics, Penzberg, Germany). For luciferase assays, cells were cotransfected with Gal4-Elk and Gal4-luc reporter plasmids [24]. After one day, cells were starved of serum for 24 hours prior to lysis. GTP-bound RAS was then isolated on RAF-RBD affinity beads as described [25]. RAS-GTP was eluted, separated by SDS-PAGE and detected with anti-HA antibody. Total HA-tagged RAS as well as ERK and phospho-ERK (Cell Signaling Technology Inc., Danvers, Massachussets) levels in cell lysates were also detected by western blot. Alternatively, luciferase activity was measured as previously described [24] using Promega reagents (Promega, Madison, Wisconsin). RAS expression was confirmed by western blot of cell lysates. Assays were performed in duplicate (RAS/ERK activity) or triplicate (luciferase) and all results independently confirmed at least 3 times.

## Results

### Clinical data

**Fig 1:**
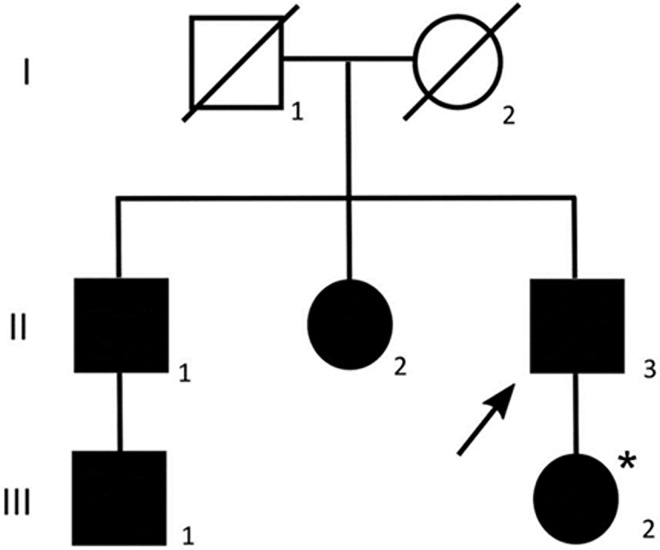
Pedigree showing proband’s family. Squares and circles symbolize males and females, respectively. Closed symbols denote subject presenting with left ventricular hypertrophy. Slashed symbols indicate deceased subjects with unknown genotype and clinical status. The proband is indicated by an arrow. The asterisk indicates the presence of intellectual disability.

The proband (Fig 1, II.3) was initially evaluated at age 60 for severe cardiac arrhythmia. Echocardiographic study revealed mild LVH and an implantable cardioverter defibrillator (ICD) was implanted as primary prevention. After 2 years the patient was hospitalized for frequent ICD discharges. Transthoracic echocardiogram showed a severe left ventricular outflow tract (LVOT) obstruction with interventricular septum thickness of 34 mm and a peak instantaneous outflow gradient of 55 mmHg under basal conditions. The patient was severely symptomatic for dyspnea, fatigue and palpitations (NYHA class III). He presented a mild mitral regurgitation with systolic anterior motion, while the systolic function was preserved (ejection fraction 70%). After surgical myectomy (4 grams) [26], interventricular wall thickness was 18 mm, the estimated gradient was reduced to 7 mmHg and no mitral abnormalities were detected. The patient’s NYHA class improved from III to I after surgery.

**Fig 2:**
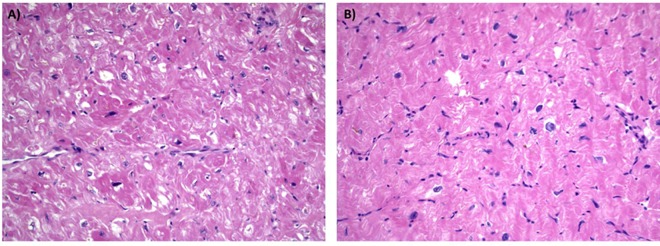
Histological analysis of myocardial samples. Comparison of interventricular septum specimens of proband and his sister. No major differences are detectable. (Hematoxylin-eosin stained section, 20X original magnification).

Histological analysis of the surgical specimen showed myocyte hypertrophy, mild myocardial fiber disarray and thickened intramural coronary arteries (Fig 2). Family history was remarkable since the proband’s nephew (Fig 1, III.1) was diagnosed with HCM at 17 years in another institute, where he was found to carry a reported MYH7 mutation (NM_000257.2: p.Met932Lys) [27]. We were informed that he was treated with beta-blockers while his father (Fig 1, II.1) presented a mild LVH that did not require any pharmacological treatment. Moreover, the proband’s sister (Fig 1, II.2) had a severe obstructive HCM that required surgical myectomy 5 years before. The proband’s daughter presented at 37 years with a mild asymptomatic LVH and severe intellectual disability. Neither the proband’s siblings nor his daughter had undergone genetic analysis at that time.

### Genetic analysis

**Fig 3:**
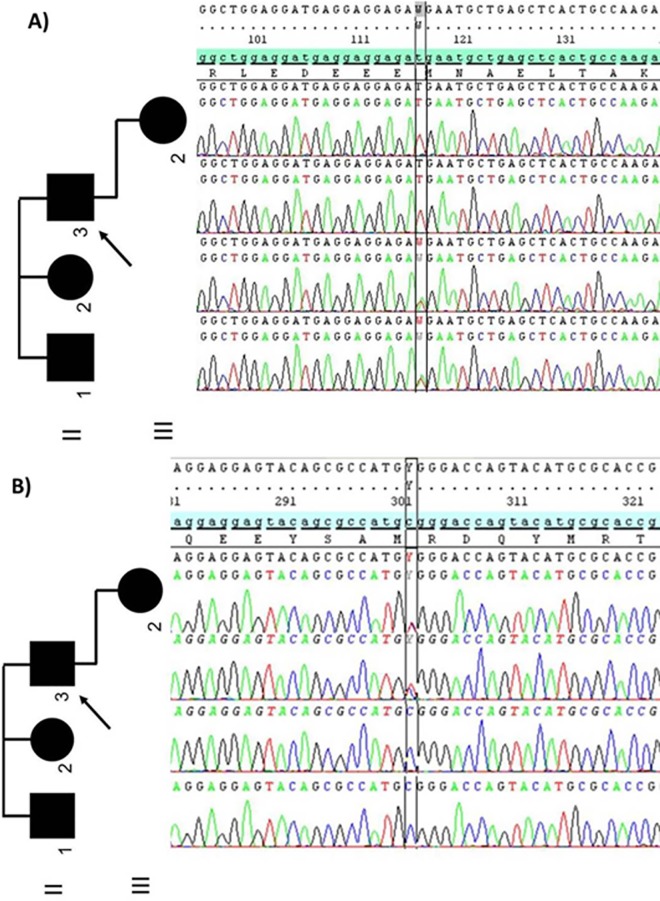
Electropherograms of the *MYH7 and HRAS* mutations in the family. Sanger sequence analysis of family members reveals A) a transversion of nucleotide NM_000257.2: c.2795T>A in *MYH7* gene in brother and sister of proband (II.1 and II.2) and B) a transition NM_005343.2:c.202C>T in *HRAS* gene in proband (II.3) and in his daughter (III.2). The pedigree is also shown.

The proband, his daughter and his siblings were analyzed by Sanger sequencing for the presence of the *MYH7* c.2795T>A mutation in exon 23. The mutation was not found either in the proband or in his daughter, while both his brother and his sister carried the *MYH7* mutation (Fig 3A).

Exome sequencing of 159 cardiac genes in the proband produced 15,524,627 mapped reads, with mean coverage of 821X and 98.7% of the target region covered at least 10X. After bioinformatic analysis, 2,744 variants in targeted genes were identified, of which 307 were in coding regions. Among exonic variants, 64% were synonymous and 98% have already been reported in the 1000 Genomes Project, ESP database or ExAC or dbSNP release 137. Among variants with MAF < 0.01%, only one heterozygous missense mutation in the *HRAS* gene (NM_005343.2:c.202C>T) was novel (MAF = 0) and remained as a plausible candidate at the end of data filtering. This Arg68Trp amino acid change is predicted to be deleterious by SIFT, MutationTaster and PolyPhen-2. Sanger sequencing was performed to confirm the presence of the variant in the proband and to assess the segregation in all available family members. We identified the *HRAS* mutation only in the proband and his daughter, who were both wild-type for the *MYH7* mutation, while the remaining family member who carried the *MYH7* mutation were negative for the *HRAS* variant (Fig 3B).

### Prediction of the variant effect by molecular dynamics simulation

**Fig 4:**
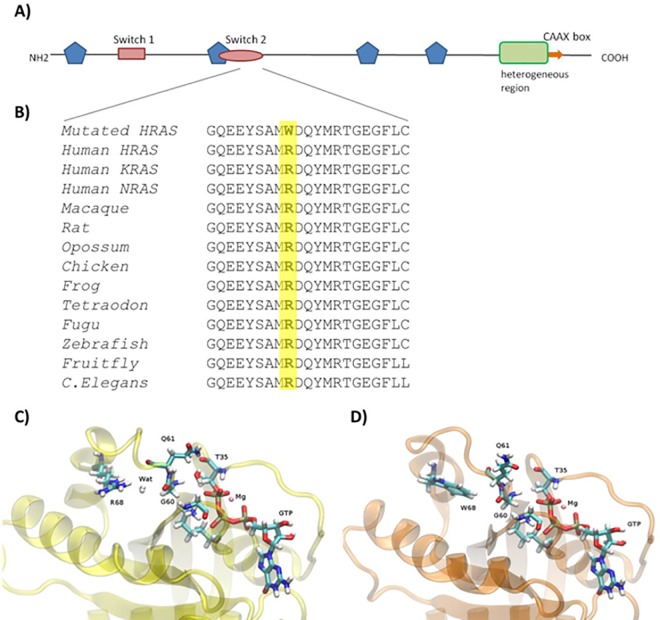
Structural analysis of HRAS protein. A) Schematic representation of HRAS structure, showing functional domains. Multiple regions participating in GTP binding are represented by blue pentagons. The switch I domain (red rectangle) is responsible for GAP interaction as well as some effector interactions, while the switch II domain (red oval) interacts with GEF. The green C-terminal box demarcates the hyper-variable regions and orange arrow demarcates CAAX box. B) Multiple-sequence alignment of Ras proteins among species is represented. The residue in position 68 is highlighted in yellow. C) and D) represent structural comparison of the most predominant conformation of HRAS wild-type and HRAS-Arg68Trp respectively from molecular dynamics simulations. In HRAS wild-type (B) the water molecule between the Arg68 and Gly60 is highlighted. In the mutant protein model (C) water molecule is weakly present.

The amino acid change (p.Arg68Trp) falls in the switch II domain of HRAS protein, which plays a key role in the GTP-mediated conformational change and RAS downstream signaling [28]. This domain is highly conserved among all the proteins belonging to the RAS superfamily [29] and among different species (Fig 4).

Molecular dynamic simulations on HRAS wild-type and HRAS-Arg68Trp in complex with GDP and GTP respectively showed a relatively stable structure with the Cα root-mean square deviations, as calculated throughout the whole simulation, in a range of 0.1 to 0.2 nm from the initial structures (S1 Fig). Structural comparison of the HRAS-wt and mutant is shown in Fig 4C and 4D. HRAS-wt has similar affinity towards GDP and GTP respectively and guanine nucleotide exchange factors (GEFs) are responsible for release of both nucleotides. Binding free energy calculations showed that the amino acid substitution strongly stabilizes the HRAS-GTP complex and destabilizes the HRAS-GDP complex, suggesting that the mutation maintains HRAS in the active GTP-bound form. Molecular dynamic studies showed that the Arg68Trp mutation involved a change of polarity in the Switch II region of HRAS protein, leading to disruption of important intramolecular interactions (S2 Fig). In particular, the crucial interaction between Arg68 and Gly60 residues through a water molecule in the GTP bound form is lost in the mutated HRAS-GTP complex. Moreover, the change of intramolecular interactions induced a change of flexibility between residues 60–80 (S3 Fig). The rearrangement of the Switch II region, an important event in order for intrinsic or GEF-mediated release of GDP and loading with GTP, seems to be restricted and impaired due to the Arg68Trp mutation. This is further supported by the binding free energy calculations under MM/GBSA approximation, as reported in Table 1.

**Table 1:** Binding free energy values calculated under MM/GBSA approximation.

Complex system	HRAS-wild-type (kcal/mol)	HRAS-Arg68Trp (kcal/mol)
HRAS-GDP	-166 ± 28	-143 ± 8
HRAS-GTP	-165 ± 16	-216 ± 7

### Analysis of mutant HRAS activity

**Fig 5:**
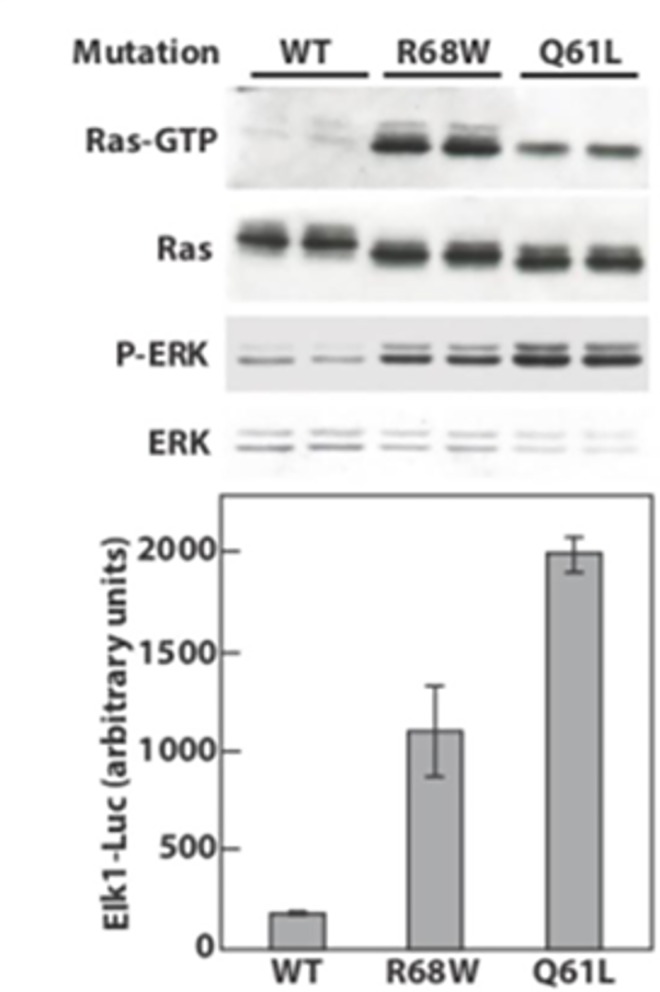
HRAS Arg68Trp mutation promotes GTP loading and activation of downstream signaling events. HEK 293T cells transfected with plasmids encoding the indicated HA-tagged Ras mutants were serum starved for 24 hr prior to lysis and detection of HA-tagged Ras, and endogenous ERK or phosphor ERK. Ras-GTP was isolated using GST-Raf-RBD-immobilized beads as detailed in Methods. Luciferase activity was measured from similarly-treated cells following cotransfection with reporter plasmids. Ras expression and ERK activation were confirmed by western blot of lysates used for luciferase activity (not shown).

To confirm *in silico* results and to measure the impact of mutation on HRAS activity, an Arg68Trp change was introduced into the *HRAS* cDNA and the ability of the encoded HRAS protein to load with GTP and to activate downstream signaling events was evaluated. HA-tagged HRAS-Arg68Trp was expressed at similar level to HRAS-wt and a previously characterized oncogenic mutant, HRAS-Gln61Leu [30] in mammalian cells, suggesting that the Arg68Trp mutation has minimal impact on protein stability. However, when the GTP-bound proteins were extracted from cell lysates using the RAS binding domain (RBD) of its effector protein, RAF, the Arg68Trp mutant was most efficiently precipitated, confirming a higher level of GTP loading than oncogenic HRAS (Fig 5).

Since the pathogenetic mechanism of HRAS is usually a consequence of activating the RAF-MEK-ERK kinase cascade, resulting in the induction of downstream gene expression by the ELK1 transcription factor [5], we directly measured ERK phosphorylation and the downstream induction of gene expression from an ELK1-responsive luciferase reporter construct. As shown in Fig 5, the ability of mutated HRAS to activate both ERK and ELK1 was intermediate between that of wild type and oncogenic HRAS, suggesting that this constitutional activation drives the cell proliferation in left ventricular hypertrophy.

## Discussion

Hypertrophic cardiomyopathy is the most common inherited cardiac condition characterized by LVH and represents an important cause of sudden cardiac death and, for this reason, it is usually considered early in the diagnostic work-up of unexplained LVH [4]. Besides HCM and its clinical phenocopies, several rare syndromic conditions can present with LVH, particularly RASopathies, which include as phenotypic features LVH and intellectual disability, as well as characteristic dysmorphic craniofacial features, failure to thrive, musculoskeletal and ectodermal abnormalities [5]. These developmental disorders are most frequently diagnosed during infancy on the bases of other major clinical manifestations, thus usually are not considered in evaluation of apparently isolated LVH, especially in adults.

Here we show the coexistence of two different genetic etiologies for LVH in the same family. We identified a novel missense mutation in the *HRAS* gene (NM_005343.2: c.202C>T) in a 62-years-old patient with severe LVOT obstruction and family history of HCM caused by a *MYH7* mutation (NM_00257.2: c.2795T>A).

Germline mutations in *HRAS* gene have been previously reported in individuals affected by Costello syndrome. It is known that 90% mutations of HRAS that contribute to RASopathies typically cluster to codons 12 and 13 and promote phenotypes that are detectable in infancy. Other rarer HRAS changes have been previously reported, affecting amino acids 58 (Thr58Ile) [31], 63 (Glu63Lys) [32] and 89 (Ser89Cys) [33]. All published cases resulted from *de novo* mutations presenting in pediatric age, except for the paternally transmitted Thr58Ile mutation, associated with a mild phenotype in adulthood. In addition, a missense mutation affecting the codon 68 (p.Arg68Gln) has been reported as likely pathogenic in ClinVar database and it is present with a low allele frequency (1/121,004) in ExAC database. Moreover mutation of KRAS residues 58–65 have been reported to increase RAS-MAPK signaling in patients with RASopathies [34, 35, 36].

Our proband (Fig 1, II.3) presented severe symptomatic LVH at an advanced age, leading to the clinical diagnosis of HCM. Furthermore, he had a positive family history for classical HCM, caused by sarcomere gene mutation. The cardiac phenotype was indistinguishable between the proband and his sister, who both underwent surgical myectomy. Moreover, no histopathological differences were detected in surgical specimens. After genetic testing, a more accurate clinical evaluation showed in the proband a mild mental impairment and some features of RASopathy such as short stature, epicanthal folds, short neck and dark skin pigmentation. The proband’s daughter (Fig 1, III.2), who carried the same HRAS mutation, presented asymptomatic LVH and severe intellectual disability, as well as typical signs of RASopathies, including short stature, micrognathia, low-set ears with short neck, epicanthal folds and ptosis.

Molecular dynamics study revealed that amino acid change from arginine to tryptophan determined a change of polarity in the Switch II region of HRAS protein, leading to the loss of molecular interaction between Arg68 and Gly60 residues in the GTP bound complex. This results in impaired rearrangement of the Switch II region, which is essential for the conformational conversion between the GDP- and GTP-bound states of HRAS. Moreover, the computed binding free energy under MM/GBSA approximation, which is inversely related to binding affinity, decreases in mutated HRAS-GTP complex (from -143 kcal/mol to -216 kcal/mol). While wild type HRAS shows similar binding free energy in complex with GDP and GTP, the mutated HRAS in complex with GDP is strongly destabilized with respect to the GTP nucleoside indicating that the latter is the predominant complex in solution.

*In silico* predictions were confirmed by functional validation *in vitro*. Indeed data collected from serum-starved cells demonstrated that HRAS-Arg68Trp exists more in the active GTP-bound state than the highly transforming Gln61Leu mutant. However consistent with previously reported mutations that contribute to Costello syndrome and other RASopathies [37], the Arg68Trp mutant activated the downstream Raf-MEK-ERK-Elk1 signaling pathway to a level intermediate between wild-type and oncogenic HRAS-Gln61Leu. While HRAS-Arg68Trp was less biologically active than the highly oncogenic Gln61Leu mutant it surprisingly bound very efficiently to the RAF-RBD (considered a measure of HRAS-GTP). However, it is well established that while different RAS mutants have similar GTPase activities in a test tube, some are more oncogenic *in vivo* [38]. Burhman et al. [39] suggest that this is due to the ability of wild-type and weakly-transforming RAS mutants to hydrolyze GTP when bound to RAF. Mutation of HRAS Gly60, a residue that normally contacts Arg68 and promotes a mild form of Costello syndrome, was recently reported to promote efficient binding to Raf but not to activate it [40]. This and a similar dominant inhibitory KRAS Gly60Arg mutation [35] suggest that suppression of growth factor signaling rather than constitutively elevated ERK activity might contribute to RASopathy. Although downstream signaling from the Arg68Trp mutant is not as strong as predicted from Raf-RBD binding, MEK and Elk1 activities are still considerably elevated by HRAS Arg68Trp under serum-starved conditions compared to wild type RAS.

In conclusion, the present study reports two different mutations in MYH7 and HRAS genes that independently contribute to left ventricular hypertrophy in the same family. This is an example of the utility of a comprehensive and deep genetic and functional analysis for the precise characterization of patients with atypical presentations of known genetic diseases, allowing a prompt diagnosis and appropriate clinical management.

## Supporting Information

S1 Fig RMSD of Cα atoms from their initial positions for the four systems. The four systems are stable for the whole simulation length.
(TIFF)

S2 Fig Schematic network contact for the four systems. The size of the sphere is proportional to the number of contact. Arg68 residues is the larger sphere in all cases. The contact is defined if any atoms of two residues are close than 0.3 nm.
(TIFF)

S3 Fig RMSF of Cα atoms from their time-averaged positions for the four system. Differences of RMSF of Cα atoms from their time-averaged positions for the four system: (A) GTP wild-type -GTP mutated, (B) GDP wild-type—GDP mutated, (C) GTP wild-type—GDP wild-type and (D) GTP mutated—GDP mutated.
(TIFF)
