# Supplementary figures and images for: A Novel HRAS Mutation Independently Contributes to Left Ventricular Hypertrophy in a Family with a Known MYH7 Mutation
Source: PLoS One. 2016 Dec 21;11(12):e0168501. doi: 10.1371/journal.pone.0168501 (PMC5176172; doi:10.1371/journal.pone.0168501)

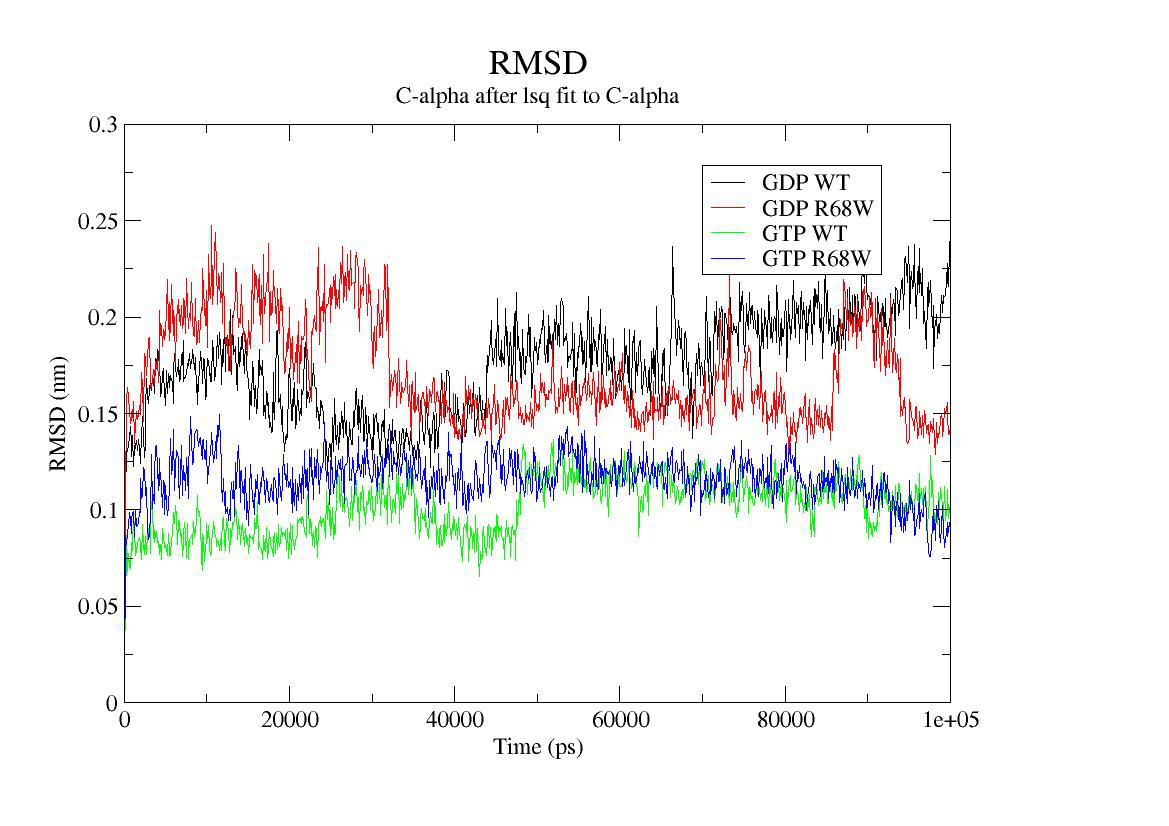

Supplement: S1 Fig — The four systems are stable for the whole simulation length. (TIFF) [file pone.0168501.s001.tiff]

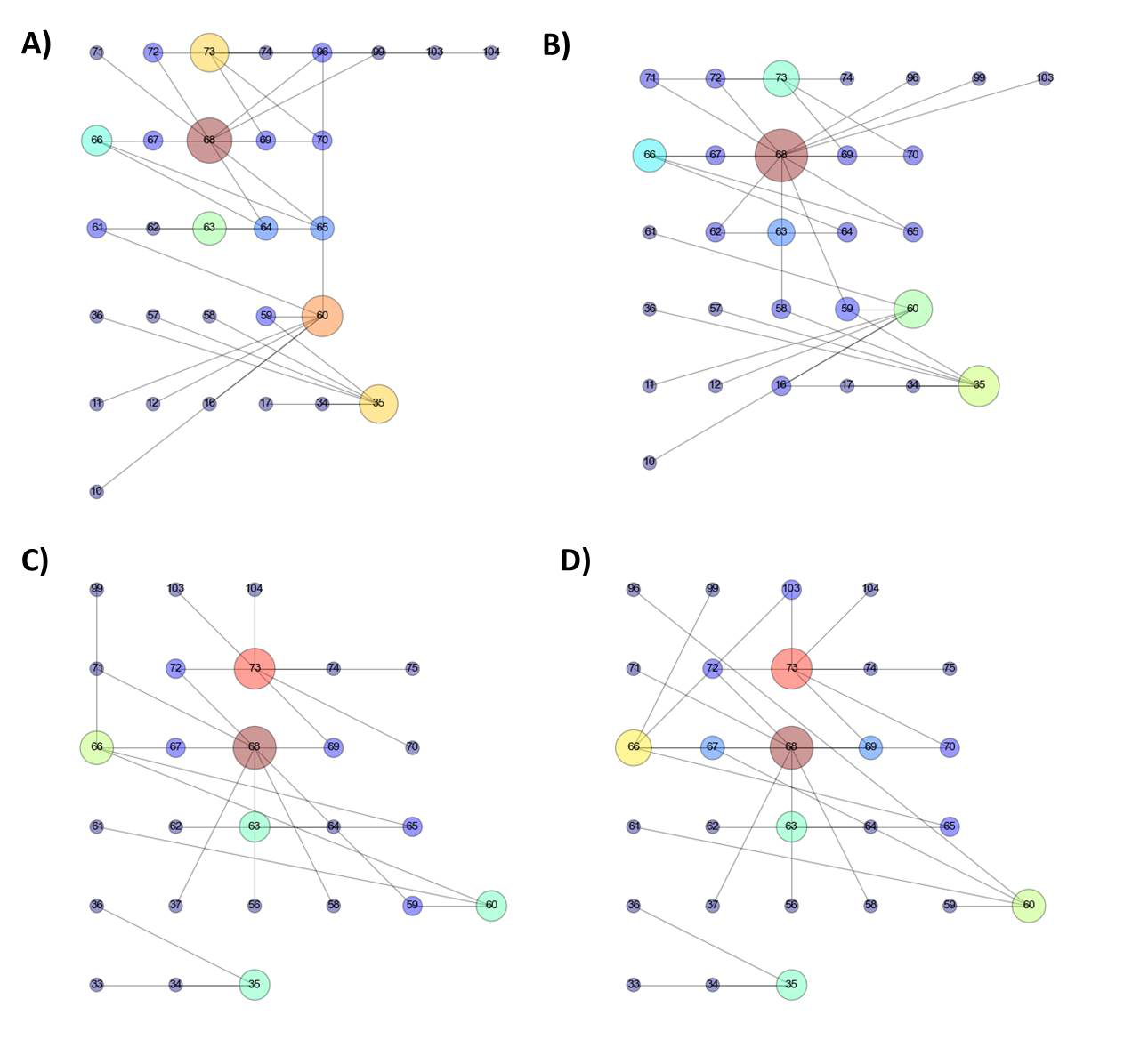

Supplement: S2 Fig — The size of the sphere is proportional to the number of contact. Arg68 residues is the larger sphere in all cases. The contact is defined if any atoms of two residues are close than 0.3 nm. (TIFF) [file pone.0168501.s002.tiff]

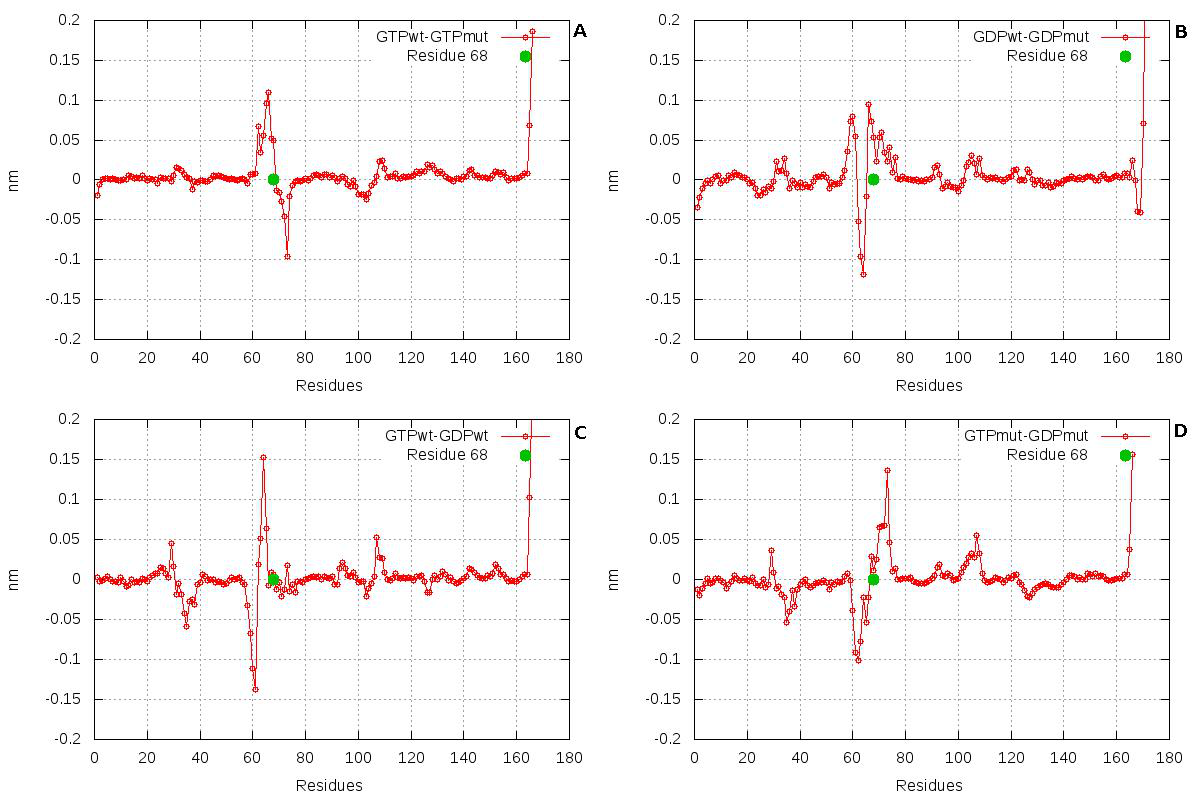

Supplement: S3 Fig — Differences of RMSF of Cα atoms from their time-averaged positions for the four system: (A) GTP wild-type -GTP mutated, (B) GDP wild-type—GDP mutated, (C) GTP wild-type—GDP wild-type and (D) GTP mutated—GDP mutated. (TIFF) [file pone.0168501.s003.tiff]
